# Partial Resection Versus Preservation of the Middle Turbinate in Endoscopic Sinus Surgery: A Systematic Review and Meta-Analysis

**DOI:** 10.3390/jcm15031288

**Published:** 2026-02-05

**Authors:** Ebraheem Albazee, Raisa Chowdhury, Dhari Altaher, Ahmed Abu-Zaid, Samer Fakhri

**Affiliations:** 1 Otorhinolaryngology-Head and Neck Surgery, Kuwait Institute for Medical Specializations (KIMS), Kuwait City 13018, Kuwait; 2 Department of Otolaryngology-Head and Neck Surgery, Al-Jahra Hospital, Al-Jahra 03200, Kuwait; 3 Faculty of Medicine and Health Sciences, McGill University, Montreal, QC H3G 2M1, Canada; raisa.chowdhury@mail.mcgill.ca; 4 Kuwait Institute for Medical Specializations (KIMS), Kuwait City 13018, Kuwait; dharisami33@gmail.com; 5 Department of Biochemistry and Molecular Medicine, College of Medicine, Alfaisal University, Riyadh 12846, Saudi Arabia; 6 Otolaryngology, Kelsey-Seybold Clinic, Houston, TX 77055, USA; fakris@yahoo.com

**Keywords:** endoscopic sinus surgery, meta-analysis, middle turbinate, partial resection, synechia

## Abstract

**Background**: To compare postoperative outcomes between partial resection and preservation of the middle turbinate (MT) in patients with chronic rhinosinusitis undergoing endoscopic sinus surgery (ESS). **Methods**: A comprehensive search was undertaken across multiple major bibliographic databases, including PubMed, Scopus, Web of Science, Embase, and the Cochrane Central Register of Controlled Trials (CENTRAL). Our outcome assessment measures included postoperative complications such as bleeding, synechia formation, MT lateralization, crustations, CSF leak, orbital injury, middle meatal antrostomy obstruction, frontal recess obstruction, revision surgery rate, smell test scores, and patient-reported outcomes (PROMs). Data were pooled using STATA software as risk ratio, mean difference, or standardized mean difference. **Results**: Fifteen clinical trials involving 2037 patients were analyzed. Partial MT resection was significantly associated with reduced rates of postoperative synechiae, MT lateralization, middle meatal obstruction, and frontal recess obstruction. Rates of postoperative bleeding, crusting, CSF leak, orbital injury, and revision surgery were comparable between the partial resection and preservation groups. No significant differences were found in olfactory outcomes. While PROMs, nasal obstruction, and headaches improved with partial resection, SNOT scores and nasal discharge remained similar. **Conclusions**: Partial MT resection demonstrated a safe and effective technique during ESS. Further large-scale RCTs are warranted to confirm and extend these findings.

## 1. Introduction

Chronic rhinosinusitis (CRS) is a commonly encountered condition managed by both rhinologists and general otolaryngologists [1,2]. Symptoms such as nasal congestion, anosmia, facial pressure, and nasal discharge can significantly impair patients’ quality of life (QoL) [1,2]. For individuals who do not respond to appropriate medical therapy, endoscopic sinus surgery (ESS) may often be the next step in management [2,3]. The primary objectives of ESS include the removal of gross inflammatory disease, establishing functional drainage and aeration of the paranasal sinuses, clearance of inspissated secretions, and facilitation of postoperative topical therapy delivery [2,4,5].

Although a range of surgical techniques is available, the optimal management of the middle turbinate (MT) during endoscopic sinus surgery (ESS) remains a subject of ongoing debate, largely due to the paucity of high-quality evidence. MT resection may be performed either partially or completely, with the approach typically determined by the extent of disease and surgeon preference [6,7,8]. Traditionally, preservation of the MT has been advocated to maintain the structural integrity of the nasal cavity. Its removal has been regarded as potentially hazardous, raising concerns about postoperative complications such as secondary frontal sinusitis, disruption of essential anatomic landmarks, and increased complexity in future revision surgeries [6,9,10]. Additional reported risks associated with MT resection include alterations in nasal airflow, excessive scar formation, postoperative hemorrhage, atrophic rhinitis, and olfactory dysfunction [10,11,12].

In contrast, several authors advocate for partial MT resection, citing advantages such as improved intraoperative visualization, enhanced surgical access to the posterior ethmoid and sphenoid sinuses, and reduced incidence of postoperative synechiae [11,12,13]. Furthermore, resection may facilitate the more effective distribution of nasal irrigations and topical corticosteroids, which could potentially improve long-term outcomes by maintaining the patency of the middle meatus and frontal recess [6,13,14].

Several clinical trials have investigated the role of partial MT resection, yet the results remain conflicting and inconsistent [15,16,17,18,19,20,21,22,23,24,25,26,27,28,29]. While some studies report that partial resection improves intraoperative and postoperative access without adversely affecting olfaction or bleeding [18,19,20,21], others have found no significant difference in outcomes when comparing resection to preservation [15,23,25].

In light of the existing uncertainty and the absence of a robust, comprehensive synthesis of the current literature, this study was designed to provide high-level evidence through a systematic review and meta-analysis of clinical trials. Specifically, we aimed to compare postoperative complication rates, olfactory test outcomes, and patient-reported outcome measures (PROMs) between partial MT resection and MT preservation among patients with CRS undergoing ESS. We anticipate that this pooled analysis will offer clearer and evidence-based guidance to support clinical decision-making among otolaryngologists and rhinologists.

## 2. Methods and Materials

This systematic review and meta-analysis adhered to the Preferred Reporting Items for Systematic Reviews and Meta-Analyses (PRISMA) guidelines (Supplementary Materials S1) [30], and followed methodological standards from the Cochrane Handbook for Systematic Reviews of Interventions to ensure rigor and transparency [31]. As this study involved secondary analysis of previously published data, ethical approval was not required. The protocol was prospectively registered on PROSPERO [CRD420251075881], providing methodological transparency and preventing selective reporting.

### 2.1. Inclusion & Exclusion Criteria

The inclusion and exclusion criteria were established using the PICO approach. Studies were considered eligible if they included adult participants (aged 18 years or older) with a diagnosis of CRS, either with or without nasal polyps, who underwent ESS. Patients assigned to the intervention group underwent partial MT resection, irrespective of the extent of resection performed, whereas the control group comprised patients in whom the MT was preserved. The primary outcomes of interest were postoperative complication rates, including bleeding, synechia formation, lateralization of the MT, crusting, cerebrospinal fluid (CSF) leak, orbital injury, obstruction of the middle meatal antrostomy, frontal recess obstruction, and revision surgery rate. Secondary outcomes included smell test score and PROMs. The study design was limited to clinical trials, including randomized controlled trials (RCTs), quasi-randomized trials, and non-randomized clinical trials (non-RCTs).

Studies were not considered eligible if they examined surgical techniques other than ESS, including transsphenoidal approaches, or if ESS was performed alongside adjunctive procedures such as inferior turbinate reduction or concha bullosa decompression. Studies evaluating interventions or comparisons unrelated to partial resection of the middle turbinate—such as complete resection, medialization, or Bolgerization—were also excluded. In addition, studies that did not employ a clinical trial design were excluded, including case reports, single-arm studies, cadaveric or anatomical investigations, observational studies, review articles, conference abstracts, and letters to the editor.

### 2.2. Literature Search & Information Sources

A systematic and comprehensive search of the literature was performed using five major electronic databases—PubMed, Scopus, Web of Science, Embase, and the Cochrane Central Register of Controlled Trials (CENTRAL)—from database inception until April 2025. The following search terms were applied: (“endoscopic sinus surgery” OR “functional endoscopic sinus surgery” OR FESS OR ESS OR “sinus surgery” OR “rhinosinus surgery” OR “paranasal sinus surgery” OR sinusotomy OR antrostomy OR ethmoidectomy) AND (“middle turbinate” OR “middle turbinate resection” OR “middle turbinate removal” OR “middle turbinate reduction” OR “middle turbinectomy” OR “partial middle turbinate resection”). No limitations were imposed with respect to publication status, country of origin, or language. The complete search strategies for all databases are provided in Table S1.

To maximize the completeness of the evidence base, we additionally conducted a manual review of the reference lists of all included articles and searched clinical trial registries, specifically ClinicalTrials.gov and the World Health Organization International Clinical Trials Registry Platform (ICTRP). We also explored supplementary sources, including ResearchGate, to identify potentially relevant unpublished or ongoing studies. When required, the corresponding authors of eligible studies were contacted to request missing information or to seek clarification regarding methodological or outcome-related details.

### 2.3. Screening & Selection Process

Study selection was performed independently by two reviewers (EA and RC) using a two-step screening process. First, the titles and abstracts of all retrieved records were screened in parallel to identify studies meeting the predefined PICOS criteria, particularly regarding study design, which was often apparent at this stage. Second, the full texts of potentially eligible studies were independently reviewed to confirm adherence to the PICO framework, with specific attention to the outcomes of interest and the availability of extractable data. Any discrepancies between the reviewers were addressed through discussion until agreement was reached. An identical independent review process was applied during the risk-of-bias evaluation and statistical data assessment to maintain methodological rigor and ensure the reliability of the included evidence.

### 2.4. Assessment of Methodological Quality

The methodological quality of RCTs was evaluated using the Cochrane Risk of Bias 2 (RoB 2) tool [32]. This framework examines potential bias across five core domains: the randomization process, deviations from the intended interventions, completeness of outcome data, outcome measurement, and selective reporting of results. Based on the assessment of these domains, each trial was classified as having a low risk of bias, some concerns, or a high risk of bias.

For non-RCTs, risk of bias was assessed using the Risk of Bias in Non-Randomized Studies of Interventions (ROBINS-I) tool v2 [33]. This instrument evaluates bias across seven domains, including confounding, participant selection, intervention classification, deviations from intended interventions, missing data, outcome measurement, and selective reporting.

### 2.5. Extraction of Data & Review-Specific Endpoints

Data from the included studies were collected using a predefined and standardized extraction template. The extracted information comprised the study ID, country in which the study was conducted, study design, sample size, recruitment timeframe, details of the intervention and comparison groups, and the duration of follow-up. In addition, baseline patient characteristics were recorded, including mean age, sex distribution (male/female), relevant clinical features, the type of surgical procedure performed, and the anesthesia technique applied.

The primary outcomes were clinical endpoints related to postoperative complications. These included the incidence rates of bleeding (%), synechia formation (%), lateralization of the middle turbinate (%), crustation (%), CSF leak (%), orbital injury (%), obstruction of the middle meatal antrostomy (%), frontal recess obstruction (%), and revision surgery rate (%).

Secondary outcomes included PROMs and olfactory function. Smell function was assessed using validated tools such as the University of Pennsylvania Smell Identification Test (UPSIT) [19], and the olfactory domain of the Sino-Nasal Outcome Test (SNOT) questionnaires [1,16,29]. PROMs such as QoL, mean score of nasal obstruction, rate of nasal discharge, and rate of headache were evaluated using instruments including the SNOT-20, SNOT-22, and the 10-point Visual Analog Scale (VAS) [16,24]. All these tools follow a consistent interpretation, where lower scores indicate clinical improvement, and higher scores reflect worsening of symptoms.

### 2.6. Meta-Analysis & Data Synthesis

Statistical analyses were performed using STATA software (version 18). Dichotomous outcomes were synthesized using risk ratios (RRs), whereas mean differences (MDs) were calculated for continuous variables. When outcomes were reported using different measurement scales, standardized mean differences (SMDs) were applied. All effect estimates were presented with 95% confidence intervals (CIs) [31], and a *p*-value < 0.05 was considered indicative of statistical significance. A fixed-effects model was initially employed for data synthesis; however, a random-effects model was applied when evidence of between-study heterogeneity was observed. Statistical heterogeneity was evaluated using both the Chi-square test and the I^2^ statistic, with *p*-values < 0.10 suggesting possible heterogeneity and I^2^ values ≥ 50% indicating substantial heterogeneity [31]. To examine the stability of the pooled results, a leave-one-out sensitivity analysis was conducted by sequentially omitting individual studies and reassessing the overall effect estimates. In instances where zero-event cells were encountered, a continuity correction of 0.5 was applied. Assessment of publication bias using funnel plots or Egger’s regression test is considered unreliable when fewer than ten studies are available for an outcome, as previously described by Egger et al. [34]. Consequently, given the limited number of studies included for each outcome in the present analysis, a formal evaluation of publication bias was not undertaken.

## 3. Results

**Figure 1:**
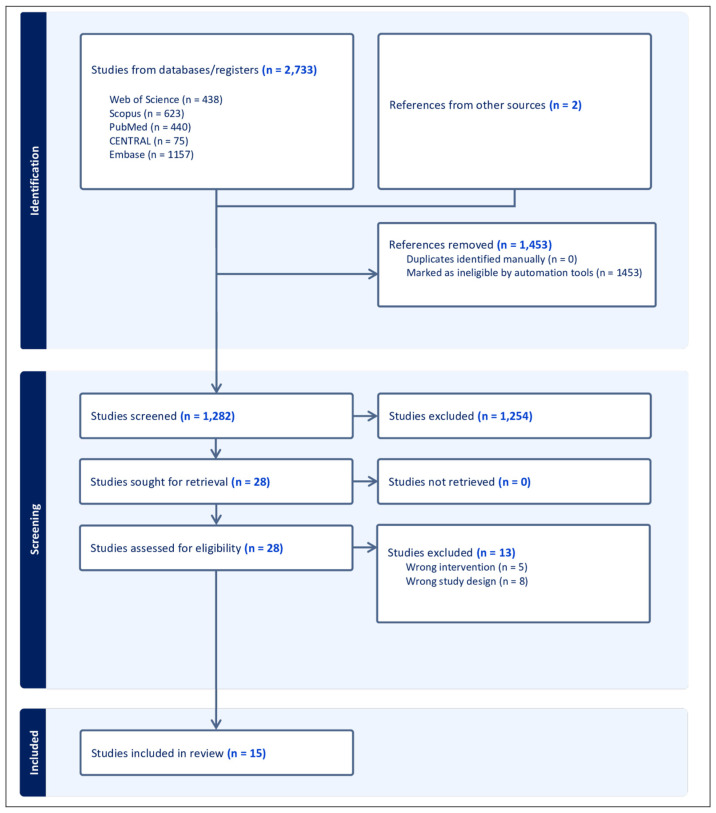
The Preferred Reporting Items for Systematic Reviews and Meta-Analyses (PRISMA) flow diagram illustrates the systematic study selection process.

The systematic literature search identified 2735 records across the included databases. Following the removal of 1453 duplicate entries, 1282 records proceeded to title and abstract screening. Subsequently, 28 full-text articles were reviewed to determine eligibility, of which 13 studies were excluded for reasons including review-type publications, evaluation of complete middle turbinate resection, cadaveric study designs, or investigations focused on Bolgerization Table S2. In total, 15 clinical trials [15,16,17,18,19,20,21,22,23,24,25,26,27,28,29] satisfied the predefined PICO criteria and were included in the final synthesis, as shown in Figure 1.

### 3.1. Summary of the Included Trials and Participants

A total of 15 clinical trials [15,16,17,18,19,20,21,22,23,24,25,26,27,28,29] were included, comprising 10 RCTs [17,18,20,21,22,25,26,27,28,29] and 5 non-RCTs [15,16,19,23,24], with a combined sample size of 2037 patients. The studies were conducted across seven countries: Egypt (*n* = 5) [15,18,23,26,29], India (*n* = 4) [20,21,27,28], Iran (*n* = 2) [17,25], and one study each from South Korea [16], the United States [19], Australia [22], and Iraq [24]. Most studies employed a parallel-group design, while four trials [15,18,20,27] used a self-controlled or intra-individual design. In the intervention arms, the majority of trials involved resection of the anteroinferior part of the middle turbinate. A detailed summary of each trial is provided in Table 1.

Regarding patient characteristics, five studies included individuals with CRS [19,20,21,22,28], one focused exclusively on CRSsNP [29], and the majority included patients with CRSwNP [15,17,18,23,24,25,26,27]. The follow-up duration across the included studies ranged from 3 months to 4.2 years, with a mean follow-up of approximately 1.02 years. Baseline characteristics of the included populations are summarized in Table 2.

The partial resection techniques of the MT across studies predominantly involved removal of the anteroinferior or anterior portion using various instruments (i.e., scissors, through-cutting forceps, microdebrider), with consistent preservation of key anatomical landmarks such as the superior sagittal segment, posterior portion, or ground lamella to minimize complications and maintain surgical orientation; the detailed MT resection techniques are presented in Table S3.

### 3.2. Summary of Methodological Quality

Risk of bias assessment revealed five RCTs with low risk [18,20,26,27,29], four with some concerns [17,21,25,28], and one with high risk [22], as summarized in Figure S1A. Delarestaghi et al. [17], Gulati et al. [21], and Santosh & Reddy [28] were judged to raise some concerns within the randomization process domain, primarily due to inadequate reporting of sequence generation and allocation concealment procedures. In contrast, Meybodian et al. [25] demonstrated some concerns related to the deviations from the intended interventions domain, as no information was provided to determine whether deviations from usual clinical practice occurred that could have impacted outcomes. Additionally, Havas & Lowinger 2000 [22] was the only study assessed as having high risk of bias, primarily due to serious concerns in the randomization process—including missing information and baseline group imbalance suggestive of allocation bias—as well as in the missing outcome data domain, since patients lost to follow-up were excluded from the final analysis.

Among the non-RCTs, two trials were assessed as having a low risk of bias [15,19], while two studies were rated as having a moderate risk of bias [16,23], and one study was judged to have a serious risk of bias [24], Figure S1B. Byun & Lee 2012 [16] received a moderate risk rating due to concerns in the selection of participants, as there was a marked imbalance between the study groups. Hussien 2013 [23] was also rated as having moderate risk due to concerns in the selection of the reported results domain, specifically for omitting certain outcomes (i.e., crustations before 3 months). The study by Jbarah & Abbas 2024 [24] exhibited a serious risk of bias, with critical concerns in both the measurement of outcomes and reporting domains.

**Table 1:** Overall summary details of the included clinical trials.

Study ID	Study Design	Country	Recruitment	Sample Size	Trial Arm		Self-Control
					Intervention	Control	
Ahmed & Osman 2016 [15]	Non-RCT	Egypt	March 2009–June 2013	*N* = 42	Partial resection of middle turbinate (anteroinferior part resected)	Preservation of middle turbinate	Yes
Byun & Lee 2012 [16]	Non-RCT	South Korea	May 2008–March 2010	*N* = 153	Partial resection of middle turbinate (inferior two-thirds to three-fourths part resected)	Preservation of middle turbinate	No
Delarestaghi et al. 2020 [17]	RCT	Iran	2017–2019	*N* = 90	Partial resection of middle turbinate (anteroinferior part resection)	Preservation of middle turbinate	No
El Antably et al. 2022 [18]	RCT	Egypt	June 2018–April 2020	*N* = 30	Partial resection of middle turbinate (anteroinferior part resected)	Preservation of middle turbinate	Yes
Friedman et al. 1996 [19]	Non-RCT	USA	NR	*N* = 64	Partial resection of middle turbinate (anteroinferior part resected)	Preservation of middle turbinate	No
Gopi et al. 2017 [20]	RCT	India	December 2014–October 2016	*N* = 30	Partial resection of middle turbinate	Preservation of middle turbinate	Yes
Gulati et al. 2010 [21]	RCT	India	NR	*N* = 40	Partial resection of middle turbinate (anterior one-third part resected)	Preservation of middle turbinate	No
Havas & Lowinger 2000 [22]	RCT	Australia	January 1987–June 1996	*N* = 1106	Partial resection of middle turbinate (anteroinferior third part resected)	Preservation of middle turbinate	No
Hussien 2013 [23]	Non-RCT	Egypt	October 2008–October 2011	*N* = 40	Partial resection of middle turbinate (anteroinferior two-thirds part resected)	Preservation of middle turbinate	No
Jbarah & Abbas 2024 [24]	Non-RCT	Iraq	November 2019–January 2021	*N* = 26	Partial resection of middle turbinate (anteroinferior part resected)	Preservation of middle turbinate	No
Lashin et al. 2023 [26]	RCT	Egypt	March 2021–August 2022	*N* = 60	Partial resection of middle turbinate (anteroinferior part resected)	Preservation of middle turbinate	No
Meybodian et al. 2025 [25]	RCT	Iran	NR	*N* = 105	Partial resection of middle turbinate (anteroinferior part resected)	Preservation of middle turbinate	No
Roy & Lade 2019 [27]	RCT	India	July 2014–January 2016	*N* = 31	Partial resection of middle turbinate (anterior two-thirds part resected)	Preservation of middle turbinate	Yes
Santosh & Reddy 2015 [28]	RCT	India	NR	*N* = 100	Partial resection of middle turbinate (anteroinferior part resected)	Preservation of middle turbinate	No
Tomoum et al. 2022 [29]	RCT	Egypt	NR	*N* = 120	Partial resection of middle turbinate (anterior part resected)	Preservation of middle turbinate	No

RCT = randomized controlled trial, NR = not reported.

**Table 2:** Baseline characteristics of the included participants and trials.

Study ID	Group	N	Age (Years)	Sex, n [Male/Female]	Patients	Surgery	Anesthesia	Follow-Up
Ahmed & Osman 2016 [15]	Partially resected MT	*n* = 42	32 ± 1	[16/26]	CRSwNP	FESS	NR	2 years
	Preserved MT	*n* = 42	32 ± 1	[16/26]				
Byun & Lee 2012 [16]	Partially resected MT	*n* = 24	39.3 (17–69)	[15/9]	CRSwNP	ESS	General	1 year
	Preserved MT	*n* = 129	41.1 (16–73)	[83/46]				
Delarestaghi et al. 2020 [17]	Partially resected MT	*n* = 45	39.73 ± 14.05	[26/19]	CRSwNP	ESS	NR	1 year
	Preserved MT	*n* = 45	39.47 ± 12.61	[28/17]				
El Antably et al. 2022 [18]	Partially resected MT	*n* = 30	33.07 ± 8.08	[14/16]	CRSwNP	FESS	General	6 months
	Preserved MT	*n* = 30	33.07 ± 8.08	[14/16]				
Friedman et al. 1996 [19]	Partially resected MT	*n* = 38	36.7 (11–68)	[29/35]	CRS	ESS	NR	1–7 months
	Preserved MT	*n* = 26						
Gopi et al. 2017 [20]	Partially resected MT	*n* = 30	38.37 ± 12.31	[17/13]	CRS	ESS	NR	6 months
	Preserved MT	*n* = 30	38.37 ± 12.31	[17/13]				
Gulati et al. 2010 [21]	Partially resected MT	*n* = 20	15–55	NR	CRS	ESS	Local	6 months
	Preserved MT	*n* = 20	15–55	NR				
Havas & Lowinger 2000 [22]	Partially resected MT	*n* = 509	15–87	[273/236]	CRS	FESS	NR	4.2 years
	Preserved MT	*n* = 597	15–87	[279/318]				
Hussien 2013 [23]	Partially resected MT	*n* = 20	39.1 (24–58)	[6/14]	CRSwNP	ESS	NR	2 years
	Preserved MT	*n* = 20	32.2 (15–60)	[12/8]				
Jbarah & Abbas 2024 [24]	Partially resected MT	*n* = 13	36 ± 13	NR	CRSwNP	ESS	General	3 months
	Preserved MT	*n* = 13	43 ± 14	NR				
Lashin et al. 2023 [26]	Partially resected MT	*n* = 30	36.4 ± 11.4	[17/13]	CRSwNP	ESS	General	9 months
	Preserved MT	*n* = 30	35.1 ± 9.9	[16/14]				
Meybodian et al. 2025 [25]	Partially resected MT	*n* = 52	NR	NR	CRSwNP	ESS	NR	6 months
	Preserved MT	*n* = 53	NR	NR				
Roy & Lade 2019 [27]	Partially resected MT	*n* = 30	NR	NR	CRSwNP	FESS	Local or general	6 months
	Preserved MT	*n* = 30	NR	NR				
Santosh & Reddy 2015 [28]	Partially resected MT	*n* = 50	15–60	NR	CRS	FESS	NR	5 months
	Preserved MT	*n* = 50	15–60	NR				
Tomoum et al. 2022 [29]	Partially resected MT	*n* = 60	40.2 ± 10.4	[75/45]	CRSnNP	ESS	General	7–13 months
	Preserved MT	*n* = 60						

MT = middle turbinate, NR = not reported, ESS = endoscopic sinus surgery, FESS = functional endoscopic sinus surgery, CRS = chronic rhinosinusitis, CRSwNP = chronic rhinosinusitis with nasal polyposis, CRSnNP = chronic rhinosinusitis without nasal polyposis.

### 3.3. Bleeding (%)

**Figure 2:**
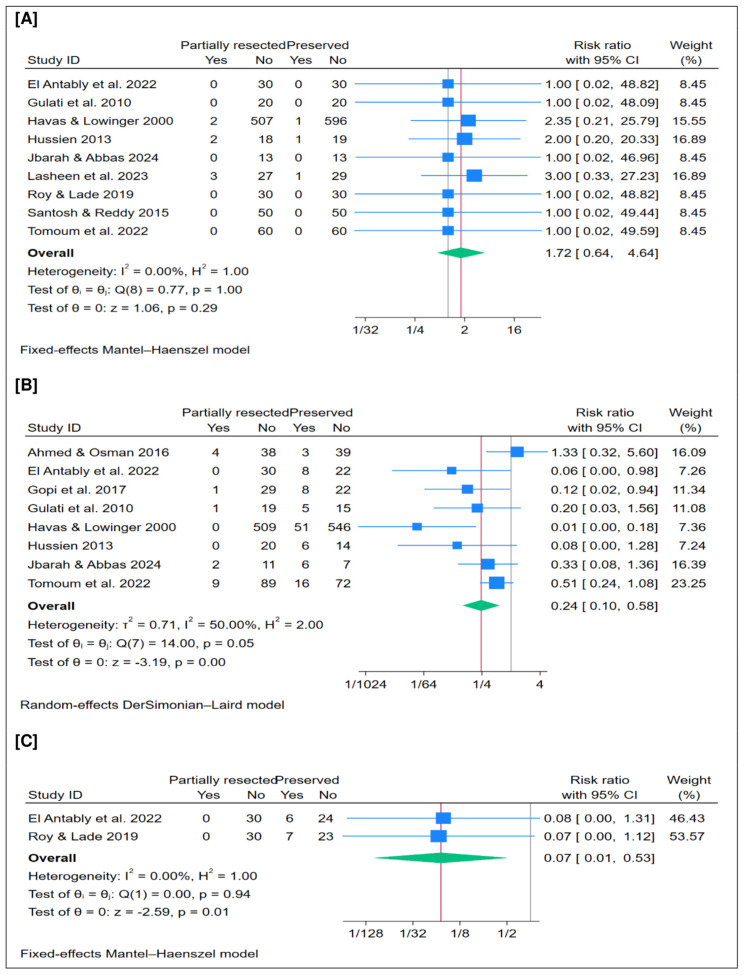
Meta-analysis of the rate of postoperative complications: (**A**) bleeding, (**B**) synechia, (**C**) lateralization of the middle turbinate. CI = confidence interval [15,18,20,21,22,23,24,26,27,28,29].

A total of 1612 patients were included in this outcome. The pooled analysis showed no significant difference in the overall postoperative bleeding rate between partial resection and preservation of the middle turbinate (*n* = 9 trials, RR = 1.72; 95% CI [0.64 to 4.64]; *p* = 0.29), as illustrated in Figure 2A. The pooled analysis demonstrated no statistical heterogeneity (I^2^ = 0%, *p* = 1.00). Moreover, leave-one-out sensitivity analysis confirmed the robustness of the findings, showing consistent results across all scenarios (Figure S2).

### 3.4. Synechia Formation (%)

A total of 1602 patients were included in this outcome. The pooled analysis showed that partial resection has a lower postoperative synechia rate compared with the preservation of the middle turbinate (*n* = 8 trials, RR = 0.24; 95% CI [0.10 to 0.58]; *p* < 0.001), as illustrated in Figure 2B. The pooled analysis demonstrated low statistical heterogeneity (I^2^ = 50%, *p* = 0.05). Moreover, leave-one-out sensitivity analysis confirmed the robustness of the findings, showing consistent results across all scenarios (Figure S3).

### 3.5. Lateralization of the MT (%)

A total of 120 patients were included in this outcome. The pooled analysis showed that partial resection has a lower rate of postoperative lateralization of the middle turbinate compared with preservation (*n* = 2 trials, RR = 0.07; 95% CI [0.01 to 0.53]; *p* = 0.01), as illustrated in Figure 2C. The pooled analysis demonstrated no statistical heterogeneity (I^2^ = 0%, *p* = 1.00). Leave-one-out sensitivity analysis was not performed since this outcome included only two clinical trials.

### 3.6. Crustations (%)

**Figure 3:**
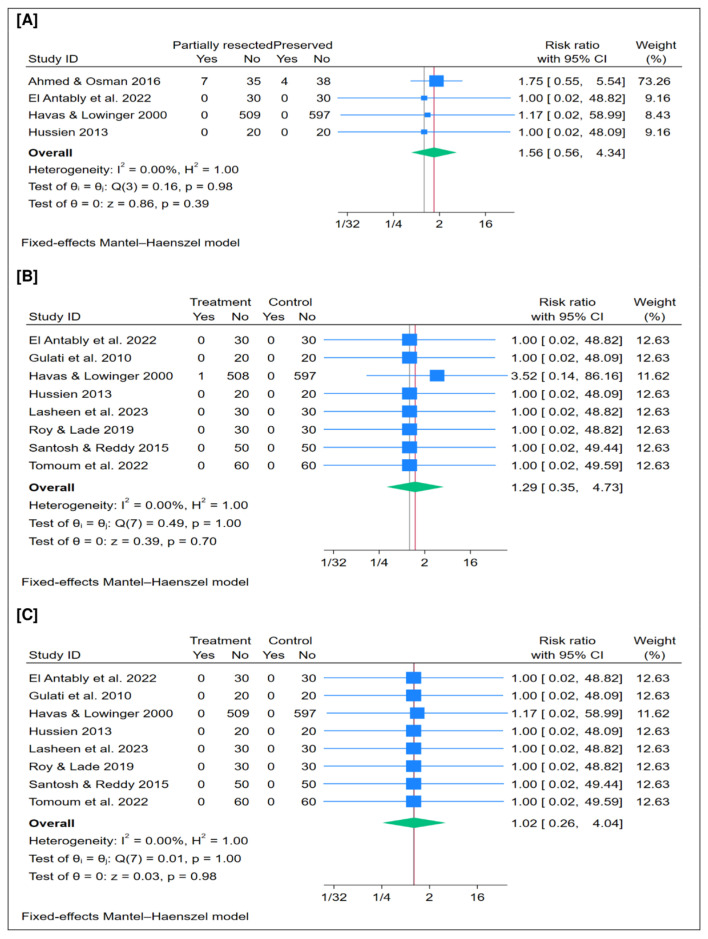
Meta-analysis of the rate of postoperative complications: (**A**) crustations, (**B**) cerebrospinal fluid (CSF) leak, and (**C**) orbital injury. CI = confidence interval [15,18,21,22,23,26,27,28,29].

A total of 1290 patients were included in this outcome. The pooled analysis showed no significant difference in the overall postoperative crustation rate between partial resection and preservation of the middle turbinate (*n* = 4 trials, RR = 1.56; 95% CI [0.56 to 4.34]; *p* = 0.39), as illustrated in Figure 3A. The pooled analysis demonstrated no statistical heterogeneity (I^2^ = 0%, *p* = 1.00). Moreover, leave-one-out sensitivity analysis confirmed the robustness of the findings, showing consistent results across all scenarios (Figure S4).

### 3.7. CSF Leak (%)

A total of 1586 patients were included in this outcome. The pooled analysis showed no significant difference in the overall postoperative CSF leak rate between partial resection and preservation of the middle turbinate (*n* = 8 trials, RR = 1.29; 95% CI [0.35 to 4.73]; *p* = 0.70), as illustrated in Figure 3B. The pooled analysis demonstrated no statistical heterogeneity (I^2^ = 0%, *p* = 1.00). Moreover, leave-one-out sensitivity analysis confirmed the robustness of the findings, showing consistent results across all scenarios (Figure S5).

### 3.8. Orbital Injury (%)

A total of 1586 patients were included in this outcome. The pooled analysis showed no significant difference in the overall postoperative orbital injury rate between partial resection and preservation of the middle turbinate (*n* = 8 trials, RR = 1.02; 95% CI [0.26 to 4.04]; *p* = 0.98), as illustrated in Figure 3C. The pooled analysis demonstrated no statistical heterogeneity (I^2^ = 0%, *p* = 1.00). Moreover, leave-one-out sensitivity analysis confirmed the robustness of the findings, showing consistent results across all scenarios (Figure S6).

### 3.9. Middle Meatus Antrostomy Obstruction (%)

**Figure 4:**
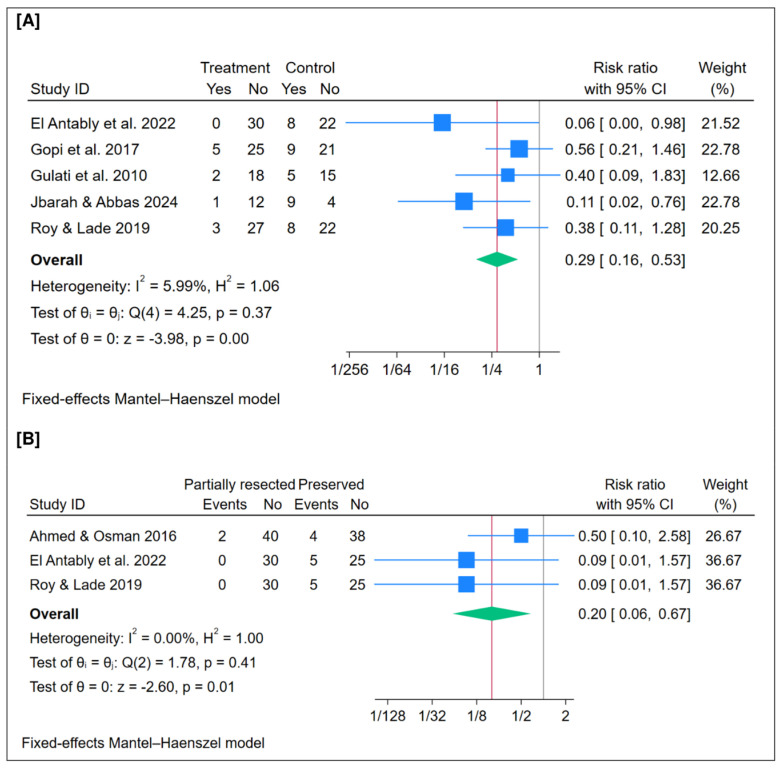
Meta-analysis of the rate of postoperative complications: (**A**) middle meatus antrostomy obstruction, and (**B**) frontal recess obstruction. CI = confidence interval [15,18,20,21,24,27].

A total of 246 patients were included in this outcome. The pooled analysis showed that partial resection has a lower postoperative middle meatus antrostomy obstruction rate compared with the preservation of the middle turbinate (*n* = 5 trials, RR = 0.29; 95% CI [0.16 to 0.53]; *p* < 0.001), as illustrated in Figure 4A. The pooled analysis demonstrated no statistical heterogeneity (I^2^ = 6%, *p* = 1.06). Moreover, leave-one-out sensitivity analysis confirmed the robustness of the findings, showing consistent results across all scenarios, Figure S7.

### 3.10. Frontal Recess Obstruction (%)

A total of 204 patients were included in this outcome. The pooled analysis showed that partial resection has a lower postoperative frontal recess obstruction rate compared with the preservation of the middle turbinate (*n* = 3 trials, RR = 0.20; 95% CI [0.06 to 0.67]; *p* = 0.01), as illustrated in Figure 4B. The pooled analysis demonstrated no statistical heterogeneity (I^2^ = 0%, *p* = 1.00). Moreover, leave-one-out sensitivity analysis showed that the pooled estimate changed to no significant difference between the two groups after excluding El Antably et al. 2022 [18], and Roy & Lade 2019 [27] (Figure S8).

### 3.11. Revision Surgery Rate (%)

**Figure 5:**
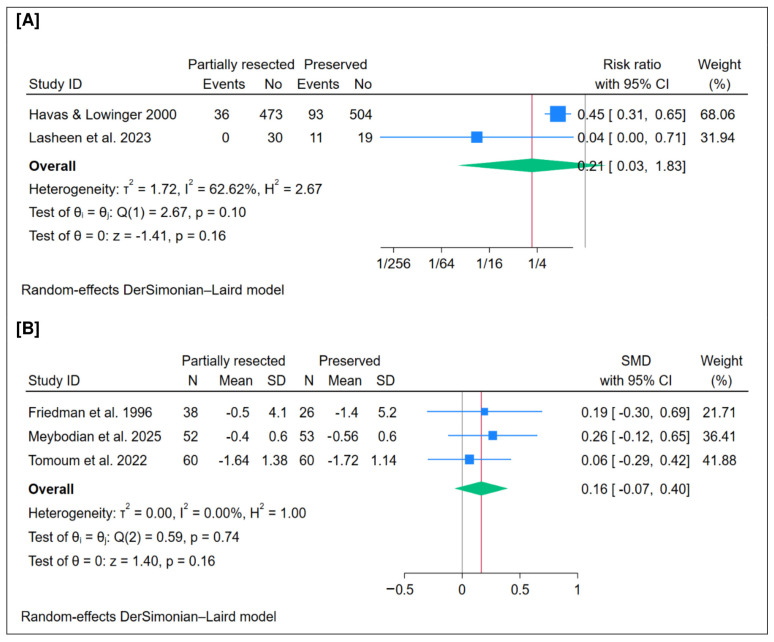
Meta-analysis of (**A**) the rate of revision surgery and (**B**) the mean smell test score. CI = confidence interval; SMD = standardized mean difference [19,22,25,26,29].

A total of 1166 patients were included in this outcome. The pooled analysis showed no significant difference in the overall revision surgery rate between partial resection and preservation of the middle turbinate (*n* = 2 trials, RR = 0.21; 95% CI [0.03 to 1.83]; *p* = 0.16), as illustrated in Figure 5A. The pooled analysis demonstrated a low statistical heterogeneity (I^2^ = 62.62%, *p* = 0.10). Leave-one-out sensitivity analysis was not performed since this outcome included only two clinical trials.

### 3.12. Mean Smell Test Score

A total of 289 patients were included in this outcome. The pooled analysis showed no significant difference in the mean smell test score between partial resection and preservation of the middle turbinate (*n* = 3 trials, SMD = 0.16; 95% CI [−0.07 to 0.40]; *p* = 0.16), as illustrated in Figure 5B. The pooled analysis demonstrated no statistical heterogeneity (I^2^ = 0%, *p* = 1.00). Moreover, leave-one-out sensitivity analysis confirmed the robustness of the findings, showing consistent results across all scenarios, Figure S9.

### 3.13. Mean SNOT Score

**Figure 6:**
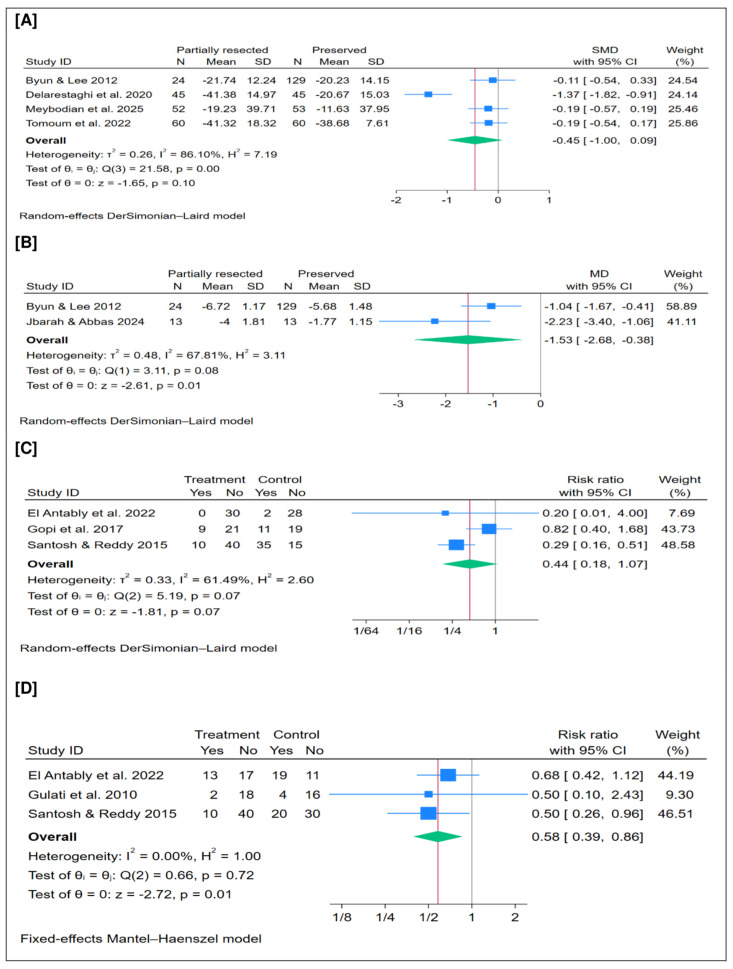
Meta-analysis of patient-reported outcomes (PROMs): (**A**) mean SNOT score, (**B**) mean nasal obstruction score, (**C**) rate of nasal discharge, and (**D**) rate of headache. SMD = standardized mean difference; MD = mean difference; CI = confidence interval [16,17,18,20,21,24,25,28,29].

A total of 486 patients were included in this outcome. The pooled analysis showed no significant difference in the mean SNOT score between partial resection and preservation of the middle turbinate (*n* = 4 trials, SMD = −0.45; 95% CI [−1.00 to 0.09]; *p* = 0.10), as illustrated in Figure 6A. The pooled analysis demonstrated no statistical heterogeneity (I^2^ = 0%, *p* = 1.00). Moreover, leave-one-out sensitivity analysis confirmed the robustness of the findings, showing consistent results across all scenarios, Figure S10.

### 3.14. Mean Nasal Obstruction Score

A total of 179 patients were included in this outcome. The pooled analysis showed that partial resection has a lower mean nasal obstruction score compared with the preservation of the middle turbinate (*n* = 2 trials, MD = −1.53; 95% CI [−2.68 to −0.38]; *p* = 0.01), as illustrated in Figure 6B. The pooled analysis demonstrated a moderate statistical heterogeneity (I^2^ = 67.81%, *p* = 0.08). Leave-one-out sensitivity analysis was not performed since this outcome included only two clinical trials.

### 3.15. Nasal Discharge (%)

A total of 220 patients were included in this outcome. The pooled analysis showed no significant difference in the nasal discharge rate between partial resection and preservation of the middle turbinate (*n* = 3 trials, RR = 0.44; 95% CI [0.18 to 1.07]; *p* = 0.07), as illustrated in Figure 6C. The pooled analysis demonstrated low statistical heterogeneity (I^2^ = 61.49%, *p* = 0.07). Moreover, leave-one-out sensitivity analysis showed that the pooled estimate changed to a significant difference between both groups that favors partial resection after excluding Gopi et al. 2017 [20], Figure S11.

### 3.16. Headache (%)

A total of 220 patients were included in this outcome. The pooled analysis showed that partial resection has a lower postoperative headache rate compared with the preservation of the middle turbinate (*n* = 3 trials, RR = 0.58; 95% CI [0.39 to 0.86]; *p* = 0.01), as illustrated in Figure 6D. The pooled analysis demonstrated no statistical heterogeneity (I^2^ = 0%, *p* = 1.00). Moreover, leave-one-out sensitivity analysis showed that the pooled estimate changed to no significant difference between the two groups after excluding Santosh & Reddy 2015 [28], Figure S12.

## 4. Discussion

This systematic review and meta-analysis comprised 15 clinical trials (*n* = 10 RCTs and *n* = 5 non-RCTs), with a total of 2037 patients comparing partial resection versus preservation of the MT in patients with CRS undergoing ESS. The patients were enrolled across seven different countries. The methodological quality of the included trials varied. The meta-analysis revealed that the partial resection group had lower rates of postoperative synechiae formation, MT lateralization, middle meatal antrostomy obstruction, and frontal recess obstruction. Both partial resection and preservation of the MT showed comparable rates of postoperative bleeding, crusting, CSF leak, orbital injury, and revision surgery. Similarly, no significant difference was found in smell test scores between the two groups. Regarding subjective assessments (i.e., PROMs), the partial resection group demonstrated lower nasal obstruction scores and a reduced rate of postoperative headache, while SNOT scores and postoperative nasal discharge rates were comparable between groups.

While most of the reported endpoints demonstrated a good degree of homogeneity, some outcomes exhibited a certain level of heterogeneity. This observed heterogeneity may be attributed to differences in patient characteristics (i.e., CRSwNP vs. CRS vs. CRSsNP), variations in methodological quality (i.e., low vs. high risk of bias), differences in follow-up duration, and heterogeneity in study design (RCTs vs. non-RCTs). In light of this heterogeneity, we performed an additional robustness analysis using the leave-one-out sensitivity method to ensure that the pooled estimates were not overly influenced by any single study. The results confirmed that the majority of the pooled outcomes were stable and consistent. However, for two endpoints—headache and frontal recess obstruction—the overall effect estimates shifted to non-significance after excluding certain studies. These specific findings should therefore be interpreted with caution.

The decision between MT preservation and resection remains a subject of ongoing debate in the context of ESS for CRS, given its potential impact on postoperative outcome and progression after ESS. The MT is a key anatomical structure involved in humidification, filtration, temperature regulation, airflow dynamics, and olfaction [13]. Despite its physiological significance, the choice to preserve or resect the MT continues to be controversial, as the literature presents conflicting evidence regarding the benefits and drawbacks of each approach. Ziegler et al. [35] conducted a survey-based study evaluating current practices of MT resection during ESS. The majority of otolaryngologists (97.6%) reported performing MT resection in specific clinical scenarios, particularly during revision surgeries [35]. Iatrogenic frontal sinus obstruction was the most concerning complication, while empty nose syndrome (ENS) was of the least concern [35]. Although debate persists, the findings indicate that most otolaryngologists are willing to perform MT resection when clinically warranted [35].

Among the most commonly reported complications following ESS are MT lateralization and synechiae formation, both of which are associated with an increased risk of revision surgery [15,18]. Synechiae, in particular, can negatively influence surgical outcomes by mechanically obstructing topical medication delivery and limiting access to the sinus cavities [36]. Furthermore, they may impair mucociliary clearance by obstructing the drainage pathways of the paranasal sinuses, such as the middle meatus and frontal recess [15,29,36].

Therefore, partial resection of the MT when warranted based on the nature and extent of disease and endoscopic anatomy may represent a viable strategy to mitigate postoperative complications. In our pooled analysis, partial MT resection was significantly associated with lower rates of postoperative MT lateralization, synechiae formation, and obstruction of the middle meatus and frontal recess. Among the included trials, the resection techniques predominantly involved removal of the anteroinferior or anterior portion of the MT using various instruments (i.e., scissors, through-cutting forceps), with consistent preservation of key anatomical landmarks—such as the superior sagittal segment or posterior attachment—to maintain stability of the middle turbinate, minimize complications and maintain surgical orientation. In addition to these approaches, various partial turbinectomy techniques have been developed to reduce turbinate bulk while preserving critical anatomy [13]. Submucosal turbinoplasty involves the removal of the turbinate bone while maintaining the overlying mucosa [37]. Medial and lateral laminectomy target hypertrophic lateral segments, offering comparable advantages and limitations to partial turbinectomy [38]. Middle turbinate medialization techniques—including transseptal sutures [39], metallic clips [40], synthetic implants [41], and nasal packing [42]—aim to improve airflow by repositioning the MT. Overall, the primary goal of these techniques is to prevent MT lateralization and reduce the incidence of synechiae and ostial obstruction.

Despite evidence supporting the benefits of partial MT resection during ESS, many surgeons remain hesitant to perform it due to concerns about potential complications such as structural instability, postoperative bleeding, CSF leak, and orbital injury [43,44]. While previous studies have reported mixed findings regarding the incidence of these complications in relation to MT resection [44,45,46], our pooled analysis demonstrated that partial MT resection (the extent of which is described in the included studies and summarized above) does not seem to increase the risk of postoperative bleeding, CSF leak, or orbital injury. Although partial MT resection is theoretically assumed to disrupt critical anatomical landmarks and increase the risk of revision surgery, our pooled analysis demonstrated comparable revision rates between the partial resection and MT preservation groups. These findings support the notion that when partial MT resection is warranted and appropriate, it does not increase the rate of revision surgery. The authors do not endorse this practice as universal in all ESS cases.

Pinna et al. (2013) conducted an anatomical study to determine the distribution of olfactory neuroepithelium in the superior and middle turbinates [47]. They found that the olfactory neuroepithelium was predominantly located in the posterior portion of the superior turbinate, regardless of side [47]. Although traces of olfactory neuroepithelium were identified in both the anterior and posterior aspects of the MT, its presence in the MT was exceedingly rare [47]. In our current meta-analysis, most included trials preserved either the posterior and/or anterior portion of the MT, and the mean olfactory test scores did not significantly differ between the MT resection and preservation groups. These findings are consistent with previous studies [8,48] suggesting that partial resection of the MT does not adversely affect olfactory function.

One of the concerns regarding MT resection is the potential development of ENS, a condition characterized by nasal dryness, crusting, and paradoxical nasal obstruction despite anatomically patent nasal cavities [8,48]. Eide et al. (2024) conducted a prospective cohort study involving 101 patients with CRSwNP to assess the risk of ENS following partial MT resection [48]. Based on multicenter data collected over a 1–2 year postoperative period, partial MT resection for CRSwNP was not associated with ENS, as none of the patients reached ENS 6-item Questionnaire (ENS6Q) scores ≥11 [48]. These findings align with those of Tan et al. (2018) [8], who also concluded that partial MT resection does not increase the risk of ENS symptoms, as defined by the ENS6Q. As such, partial MT resection can be safely considered during ESS [8]. However, it is important to note that in our systematic review, none of the included trials reported ENS as an outcome, and it was not evaluated as an endpoint.

Nasal obstruction is a common symptom of CRS, often accompanied by nasal congestion, rhinorrhea, anosmia, headache, respiratory difficulties, and posterior nasal drip [1,2]. Partial resection of the MT can alleviate this obstruction, thereby improving symptoms and enhancing patients’ QoL [1,2]. In the current meta-analysis, partial MT resection was significantly associated with reduced nasal obstruction scores and a lower rate of headache. However, no significant differences were observed in SNOT scores or postoperative nasal discharge rates compared to MT preservation. Closer inspection of Figure 6A–D (i.e., PROMs) reveals a consistent trend favoring the partial resection group, with most CIs (>90%) lying on that side. The absence of statistical significance—particularly for the SNOT score—may be attributed to the limited number of included studies and small sample size. Delarestaghi et al. (2020) [17] reported that partial MT resection with ESS improved symptoms and QoL, whereas Tomoum et al. (2022) [29] and Meybodian et al. (2025) [25] found no significant differences in QoL between resection and preservation groups. Overall, these interpretations are based on individual studies with a limited sample size, and further well-designed trials are needed to validate these findings.

In the present meta-analysis, revision rates did not differ significantly between groups. Revision surgery may be influenced by CRS phenotype (CRSwNP vs. CRSsNP) and disease severity. In this context, Wu et al. [49] reported a significantly longer time to revision surgery in patients who underwent middle turbinate resection compared with preservation (4.56 vs. 3.93 years; *p* = 0.048), with this effect persisting for up to eight years after surgery. These findings suggest a potential long-term benefit of turbinate resection in selected patients [49]. However, phenotype-specific subgroup analyses were not feasible in the present review due to inconsistent reporting across included studies. Therefore, revision rate outcomes should be interpreted with caution.

This investigation has several noteworthy strengths. It is the first study to quantitatively compare partial resection versus preservation of the middle turbinate, incorporating a relatively large sample size of 2037 patients from multiple countries worldwide. The study was conducted in accordance with PRISMA guidelines, and a comprehensive search across five major databases ensured the inclusion of all relevant literature. By restricting the analysis to clinical trials, we enhanced the methodological rigor and the reliability of our findings. Furthermore, we focused exclusively on partial resection, excluding studies on complete resection to ensure consistency in the intervention and comparator groups. Finally, a leave-one-out sensitivity analysis was performed to assess the robustness and stability of the pooled results.

However, several limitations should be acknowledged. The small number of pooled studies per outcome for certain assessment measures (i.e., revision surgery rate) limits the generalizability of the findings. Variability in patient characteristics, methodological quality, follow-up durations, and study designs (RCTs vs. non-RCTs) may have contributed to heterogeneity. In addition, all CRS phenotypes were included in the analysis, and differences between CRSsNP and CRSwNP—particularly with respect to disease behavior and revision surgery patterns—may represent a potential source of bias when interpreting surgical outcomes. Furthermore, some clinically relevant outcomes (i.e., ENS) were not reported, preventing a comprehensive assessment of the full impact of partial MT resection. Finally, with fewer than 10 studies per outcome, the assessment of publication bias was limited, reducing confidence in evaluating the influence of unpublished or selectively reported studies.

Based on the findings of this meta-analysis, several recommendations can be made for future research and clinical practice. Larger, well-designed RCTs are needed to further evaluate the efficacy of partial MT resection in patients with CRS undergoing ESS. Future studies should utilize standardized outcome measures and assess efficacy both objectively—through endoscopic and radiological evaluation—and subjectively, via PROMs such as quality of life and symptom severity. Longer follow-up durations are also recommended, along with a comprehensive assessment of olfactory function and the incidence of ENS.

## 5. Conclusions

This systematic review and meta-analysis of 15 clinical trials involving 2037 patients demonstrated that partial MT resection was significantly associated with reduced rates of postoperative synechiae, MT lateralization, middle meatal obstruction, and frontal recess obstruction. Rates of postoperative bleeding, crusting, CSF leak, orbital injury, and revision surgery were comparable between the partial resection and preservation groups. No significant differences were found in olfactory outcomes. While PROMs, nasal obstruction, and headaches improved with partial resection, SNOT scores and nasal discharge rates remained similar. Further large-scale RCTs are warranted to confirm and extend these findings.

## Data Availability

All data are available within the manuscript and can be obtained from the corresponding author upon a reasonable request.

## Supplementary Materials

The following supporting information can be downloaded at: https://www.mdpi.com/article/10.3390/jcm15031288/s1, Material S1: PRISMA checklist. Table S1: Detailed search strategy for each database. Table S2: List of excluded studies during the full-text screening step. Table S3: The detailed techniques for the partial resection of the middle turbinate. Figure S1: Risk of bias (A) (RoB-2) graph for randomized controlled trials, (B) (ROBINS-I) graph for non-randomized controlled trials. Figure S2: Leave-one-out sensitivity analysis of postoperative bleeding rate. Figure S3: Leave-one-out sensitivity analysis of postoperative synechia rate. Figure S4: Leave-one-out sensitivity analysis of postoperative crustation rate. Figure S5: Leave-one-out sensitivity analysis of postoperative CSF leak rate. Figure S6: Leave-one-out sensitivity analysis of postoperative orbital injury rate. Figure S7: Leave-one-out sensitivity analysis of postoperative middle meatus antrostomy obstruction rate. Figure S8: Leave-one-out sensitivity analysis of postoperative frontal recess obstruction rate. Figure S9: Leave-one-out sensitivity analysis of the mean smell test score. Figure S10: Leave-one-out sensitivity analysis of the mean SNOT score. Figure S11: Leave-one-out sensitivity analysis of postoperative nasal discharge rate. Figure S12: Leave-one-out sensitivity analysis of postoperative headache rate.
