# Supplementary material for: Partial Resection Versus Preservation of the Middle Turbinate in Endoscopic Sinus Surgery: A Systematic Review and Meta-Analysis
Source: J Clin Med. 2026 Feb 5;15(3):1288. doi: 10.3390/jcm15031288 (PMC12898332; doi:10.3390/jcm15031288)

TABLE AND FIGURE LEGENDS

TABLE S1. Detailed search strategy for each database.

TABLE S2. List of excluded studies during the full-text screening step.

TABLE S3. The detailed techniques for the partial resection of the middle turbinate.

FIGURE S1. Risk of bias **[A]** (RoB-2) graph for randomized controlled trials, **[B]** (ROBINS-I) graph for non-randomized controlled trials.

FIGURE S2. Leave-one-out sensitivity analysis of postoperative bleeding rate.

FIGURE S3. Leave-one-out sensitivity analysis of postoperative synechia rate.

FIGURE S4. Leave-one-out sensitivity analysis of postoperative crustation rate.

FIGURE S5. Leave-one-out sensitivity analysis of postoperative CSF leak rate.

FIGURE S6. Leave-one-out sensitivity analysis of postoperative orbital injury rate.

FIGURE S7. Leave-one-out sensitivity analysis of postoperative middle meatus antrostomy obstruction rate.

FIGURE S8. Leave-one-out sensitivity analysis of postoperative frontal recess obstruction rate.

FIGURE S9. Leave-one-out sensitivity analysis of the mean smell test score.

FIGURE S10. Leave-one-out sensitivity analysis of the mean SNOT score.

FIGURE S11. Leave-one-out sensitivity analysis of postoperative nasal discharge rate.

FIGURE S12. Leave-one-out sensitivity analysis of postoperative headache rate.

TABLE S1. Detailed search strategy for each database.

Database	Search Strategy	Filter	Results
**PubMed**	("endoscopic sinus surgery"[Title/Abstract] OR "functional endoscopic sinus surgery"[Title/Abstract] OR "FESS"[Title/Abstract] OR "ESS"[Title/Abstract] OR "sinus surgery"[Title/Abstract] OR "rhinosinus surgery"[Title/Abstract] OR "paranasal sinus surgery"[Title/Abstract] OR "sinusotomy"[Title/Abstract] OR "antrostomy"[Title/Abstract] OR "ethmoidectomy"[Title/Abstract]) AND ("middle turbinate"[Title/Abstract] OR "middle turbinate resection"[Title/Abstract] OR "middle turbinate removal"[Title/Abstract] OR "middle turbinate reduction"[Title/Abstract] OR "middle turbinectomy"[Title/Abstract] OR "middle turbinoplasty"[Title/Abstract] OR "partial middle turbinate resection"[Title/Abstract])	All Fields	N = 440
**CENTRAL**	("endoscopic sinus surgery" OR "functional endoscopic sinus surgery" OR "FESS" OR "ESS" OR "sinus surgery" OR "rhinosinus surgery" OR "paranasal sinus surgery" OR "sinusotomy" OR "antrostomy" OR "ethmoidectomy") AND ("middle turbinate" OR "middle turbinate resection" OR "middle turbinate removal" OR "middle turbinate reduction" OR "middle turbinectomy" OR "middle turbinoplasty" OR "partial middle turbinate resection")	Title Abstract Keyword	N = 75
**Web of Science**	("endoscopic sinus surgery" OR "functional endoscopic sinus surgery" OR "FESS" OR "ESS" OR "sinus surgery" OR "rhinosinus surgery" OR "paranasal sinus surgery" OR "sinusotomy" OR "antrostomy" OR "ethmoidectomy") AND ("middle turbinate" OR "middle turbinate resection" OR "middle turbinate removal" OR "middle turbinate reduction" OR "middle turbinectomy" OR "middle turbinoplasty" OR "partial middle turbinate resection")	All Fields	N = 438
**Scopus**	TITLE-ABS-KEY ("endoscopic sinus surgery" OR "functional endoscopic sinus surgery" OR "FESS" OR "ESS" OR "sinus surgery" OR "rhinosinus surgery" OR "paranasal sinus surgery" OR "sinusotomy" OR "antrostomy" OR "ethmoidectomy") AND TITLE-ABS-KEY ("middle turbinate" OR "middle turbinate resection" OR "middle turbinate removal" OR "middle turbinate reduction" OR "middle turbinectomy" OR "middle turbinoplasty" OR "partial middle turbinate resection")	Article title, Abstract, Keywords	N = 623
**Embase**	("endoscopic sinus surgery" OR "functional endoscopic sinus surgery" OR "FESS" OR "ESS" OR "sinus surgery" OR "rhinosinus surgery" OR "paranasal sinus surgery" OR "sinusotomy" OR "antrostomy" OR "ethmoidectomy") AND ("middle turbinate" OR "middle turbinate resection" OR "middle turbinate removal" OR "middle turbinate reduction" OR "middle turbinectomy" OR "middle turbinoplasty" OR "partial middle turbinate resection")	All Fields	N = 1157

TABLE S2. List of excluded studies during the full-text screening step.

Study ID	Title	Reason of exclusion
**Eide et al. 2025**	Subtotal Middle Turbinate Resection in Patients with Chronic Rhinosinusitis with Nasal Polyps is Unlikely to Cause Empty Nose Syndrome: A Multi-Institutional Prospective Study	Observational study
**Novarria et al. 2024**	Efficacy and safety of middle turbinate surgery: a systematic review	Systematic review
**Hudon et al. 2018**	Resection versus preservation of the middle turbinate in surgery for chronic rhinosinusitis with nasal polyposis: a randomized controlled trial	Complete resection
**Tan et al. 2018**	Partial resection of the middle turbinate during endoscopic sinus surgery for chronic rhinosinusitis does not lead to an increased risk of empty nose syndrome: a cohort study of a tertiary practice	Observational study
**Scangas et al. 2017**	Does the Timing of Middle Turbinate Resection Influence Quality-of-Life Outcomes for Patients with Chronic Rhinosinusitis?	Observational study
**Halderman et al. 2016**	The effect of middle turbinate resection on topical drug distribution into the paranasal sinuses	Cadaveric study
**Marchioni et al. 2008**	Middle turbinate preservation versus middle turbinate resection in endoscopic surgical treatment of nasal polyposis	Complete resection
**Su et al. 2022**	Effect of modified endoscopic sinus surgery combined with middle turbinate resection on olfactory function and stress response in patients with refractory chronic rhinosinusitis with nasal polyps	Complete resection
**Banfield & McCombe 1999**	Partial Resection of the Middle Turbinate at Functional Endoscopic Sinus Surgery	Single-arm trial
**Khafagy et al. 2021**	The impact of bolgerization versus partial resection of the middle turbinate on frontal sinusotomy patency outcome: a randomised controlled study	Bolgerization
**Law et al. 2021**	Middle turbinate resection is unlikely to cause empty nose syndrome in first year postoperatively	Observational study and single-arm
**Choby et al. 2014**	Clinical Effects of Middle Turbinate Resection after Endoscopic Sinus Surgery: A Systematic Review	Systematic review
**Mostafa et al. 2019**	Key role of inferior turbinectomy and partial middle turbinectomy in endoscopic surgery for allergic fungal sinusitis: A comparative study	Combined with ITR

TABLE S3. The detailed techniques for the partial resection of the middle turbinate.

Study ID	The detailed techniques for the partial resection of the middle turbinates
**Ahmed & Osman 2016**	Partial resection involved removing the anteroinferior part of the middle turbinate using nasal scissors or through-cutting instruments, while preserving approximately 0.5 cm of the superior sagittal portion to maintain anatomical landmarks and minimize lateralization.
**Byun & Lee 2012**	Partial resection involved removing the inferior two-thirds to three-fourths of the middle turbinate using through-cutting forceps, while preserving the superior sagittal portion as an anatomical landmark.
**Delarestaghi et al. 2020**	Endoscopic partial middle turbinectomy was performed by resecting the anteroinferior part of the middle turbinate using turbinate scissors, while preserving both the superior portion and the posterior segment as anatomical landmarks.
**El Antably et al. 2022**	Partial resection was performed using angled scissors to excise a small (~1 cm) wedge from the anterior end of the middle turbinate via two cuts—one postero-inferior and the other upward—typically at the end of the operation, or earlier if access to the osteomeatal complex was limited.
**Friedman et al. 1996**	When resection was indicated, curved endoscopic nasal scissors were used to perform it, and a stub of the vertical attachment was preserved to serve as a landmark.
**Gopi et al. 2017**	Not reported
**Gulati et al. 2010**	The anterior one-third of the middle turbinate was resected using turbinate scissors or through-cutting forceps.
**Havas & Lowinger 2000**	Partial resection was performed at the start of the procedure by removing only the anterior-inferior third of the middle turbinate using curved endoscopic scissors, following medialization and hemostasis.
**Hussien 2013**	The anteroinferior two-thirds of the middle turbinate were resected using through-cutting instruments, while preserving the superior and lateral attachments as landmarks, along with a small posterior stump near the sphenopalatine foramen.
**Jbarah & Abbas 2024**	After medialization with a Freer's elevator, the anteroinferior part of the middle turbinate was resected using turbinectomy scissors.
**Lasheen et al. 2023**	Partial middle turbinectomy involved resection of the anterior-inferior hanging portion of the turbinate to widen the middle meatus and prevent lateral displacement that could obstruct sinus outflow tracts.
**Meybodian et al. 2025**	Partial turbinectomy was performed by making an incision in the lower anterior part of the middle turbinate, with preservation of the posterior portion as a landmark for sphenoidotomy.
**Roy & Lade 2019**	The anterior two-thirds of the middle turbinate were resected, with preservation of the ground lamella.
**Santosh & Reddy 2015**	Partial middle turbinate resection was performed while preserving the superior and posterior portions to maintain key surgical landmarks. Indications included relieving middle meatus obstruction, preventing postoperative synechiae, addressing concha bullosa or polypoidal mucosal changes, and improving surgical access.

**Tomoum et al. 2022**	A vertical strip of the anterior part of the middle turbinate (anterior to the maxillary line) and its bulbous medial portion were resected using through-cutting forceps, Kerrison punch, and microdebrider, while sparing the upper part and mucosa over the axilla.

FIGURE S1. Risk of bias **[A]** (RoB-2) graph for randomized controlled trials, **[B]** (ROBINS-I) graph for non-randomized controlled trials.

[A]

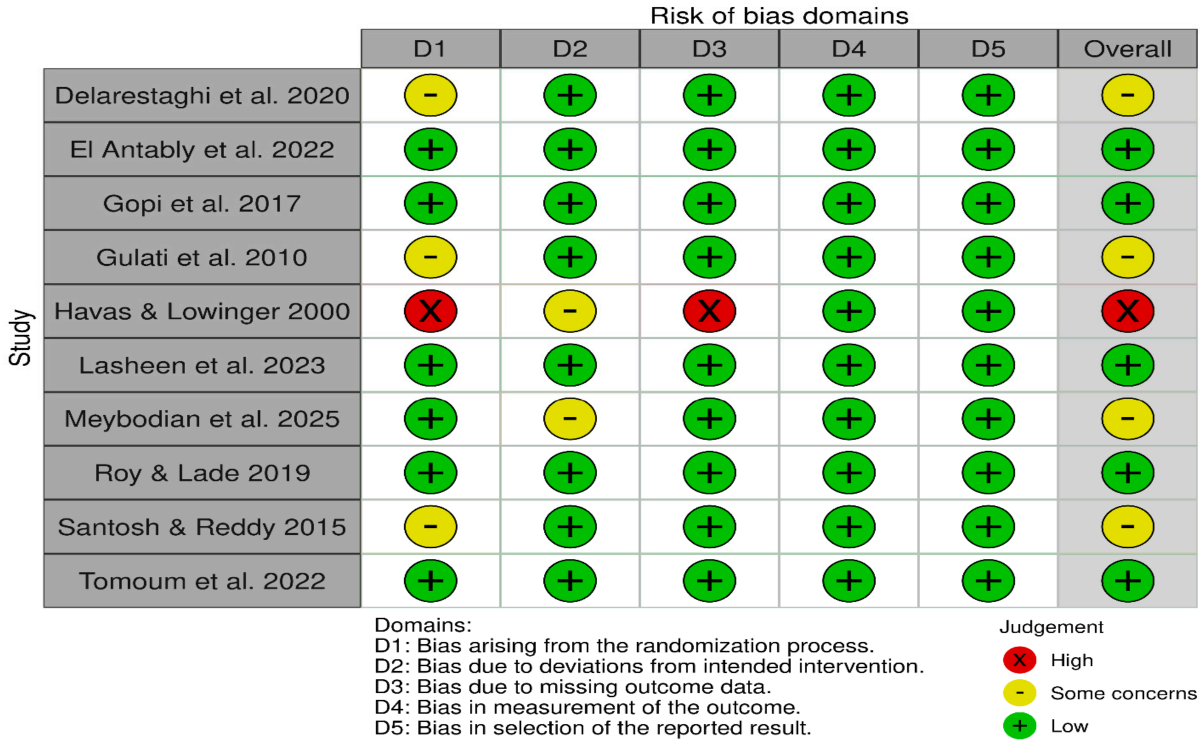

[B]

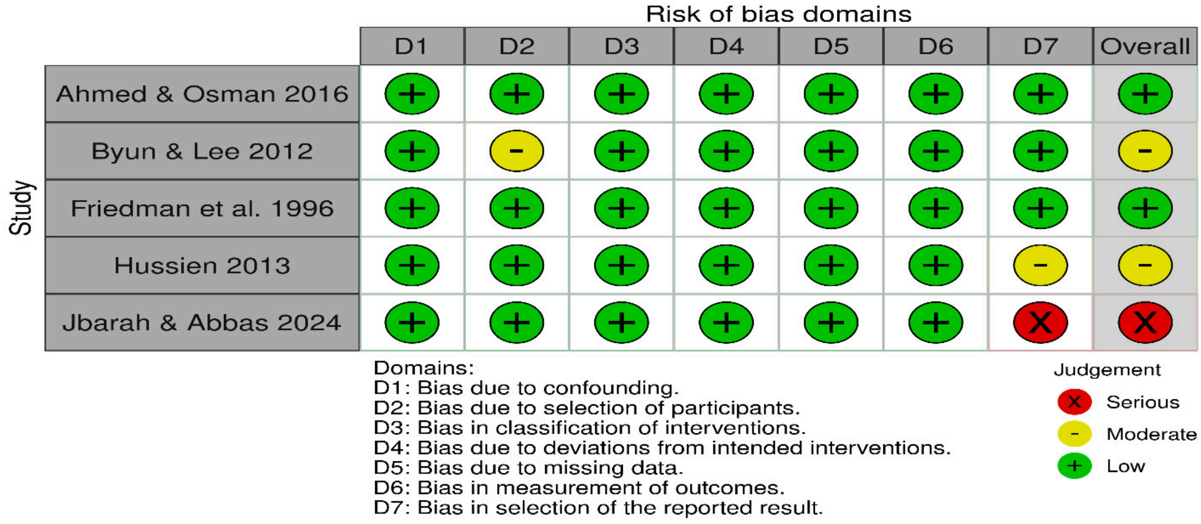

FIGURE S2. Leave-one-out sensitivity analysis of postoperative bleeding rate.

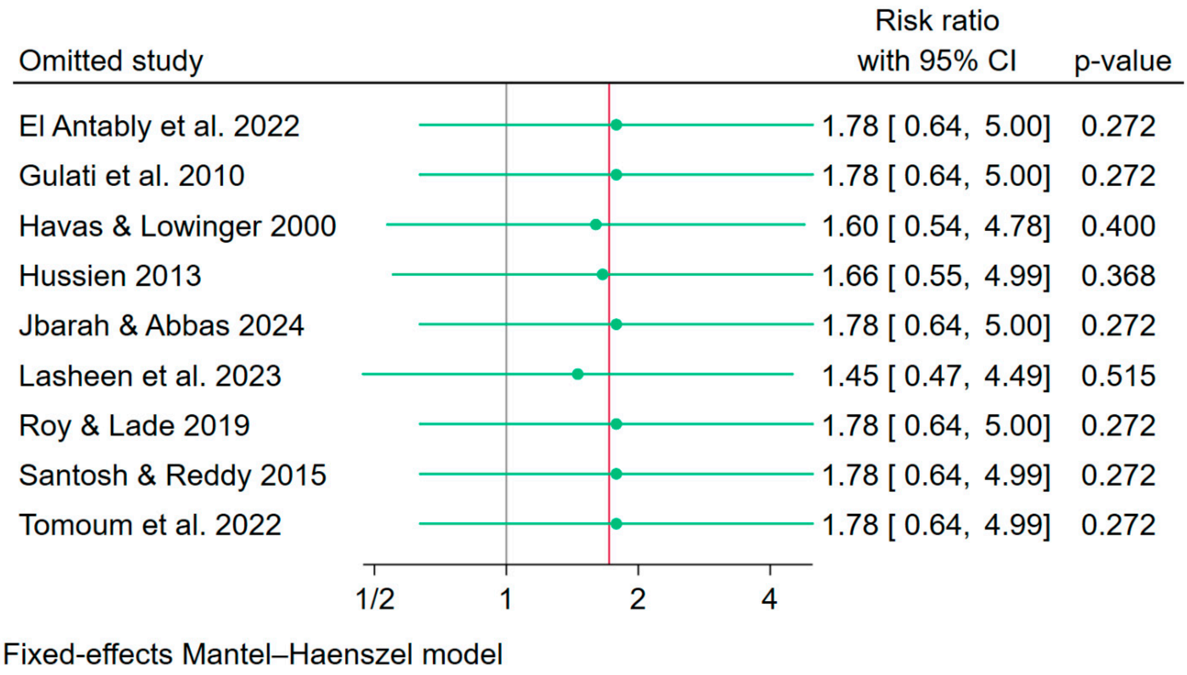

FIGURE S3. Leave-one-out sensitivity analysis of postoperative synechia rate.

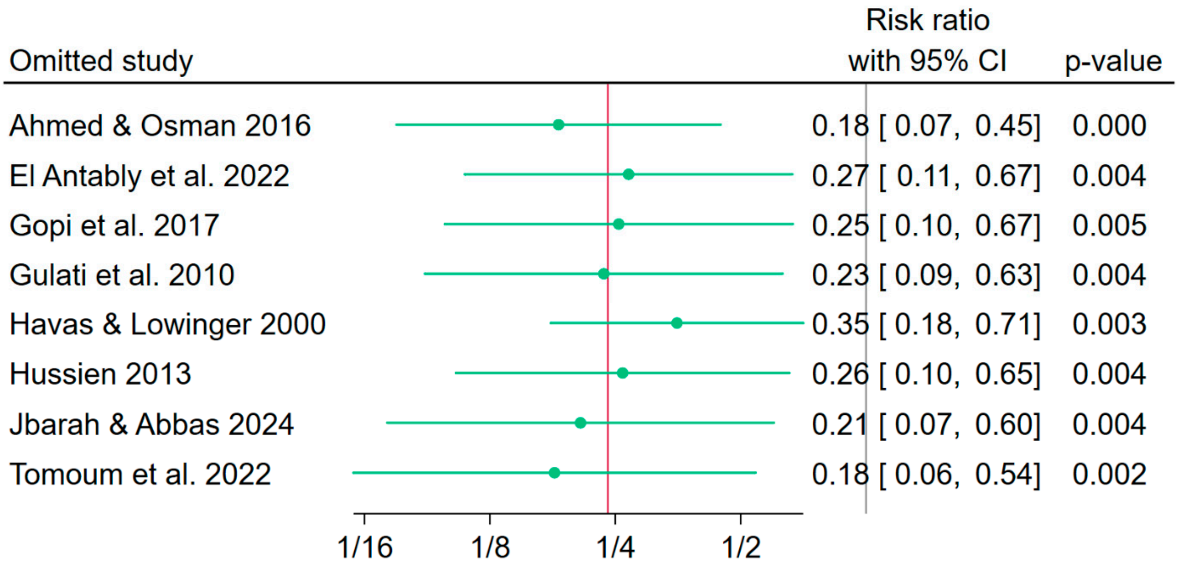

Random-effects DerSimonian–Laird model

FIGURE S4. Leave-one-out sensitivity analysis of postoperative crustation rate.

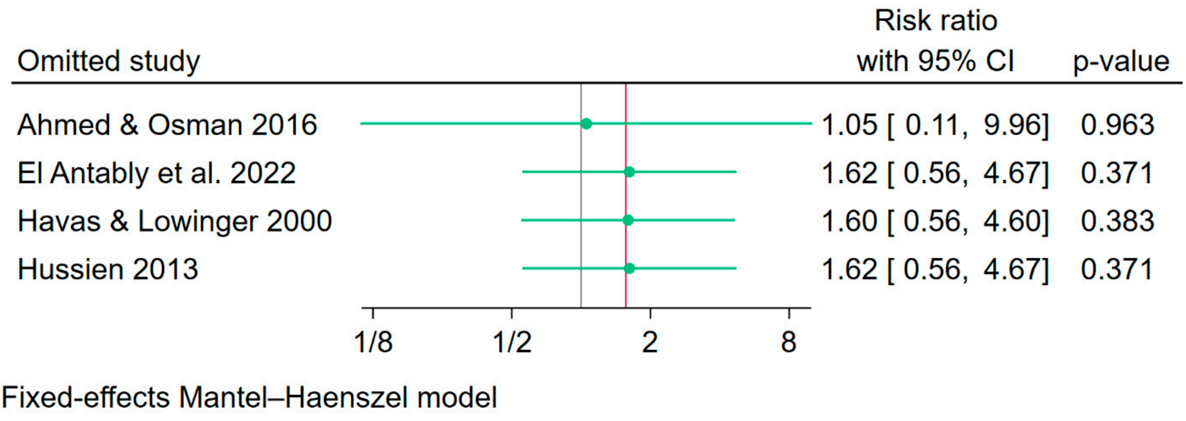

FIGURE S5. Leave-one-out sensitivity analysis of postoperative CSF leak rate.

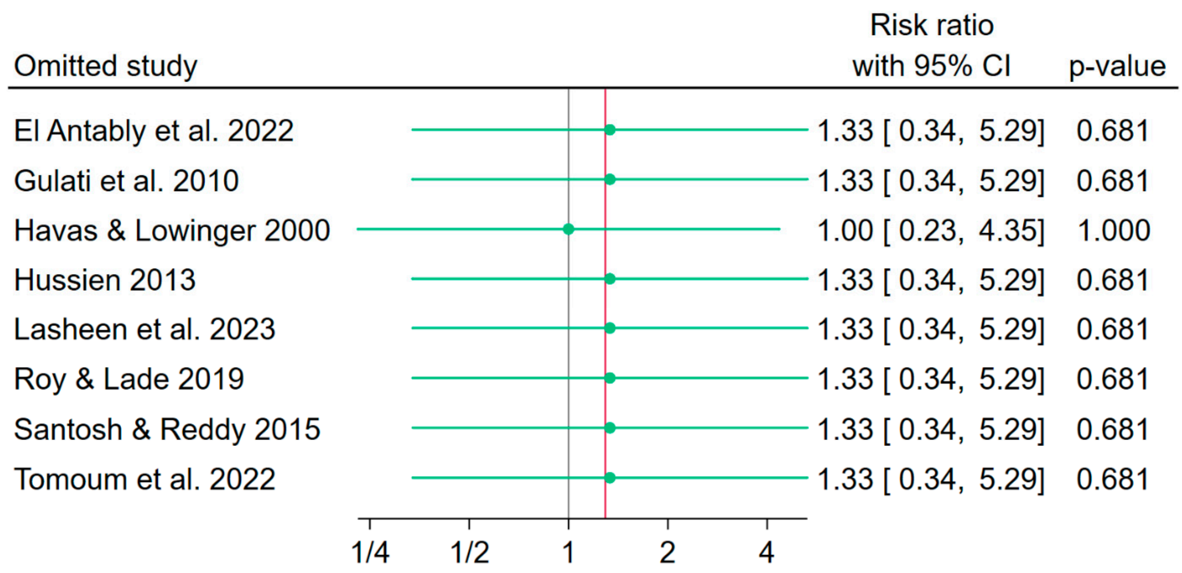

Fixed-effects Mantel–Haenszel model

FIGURE S6. Leave-one-out sensitivity analysis of postoperative orbital injury rate.

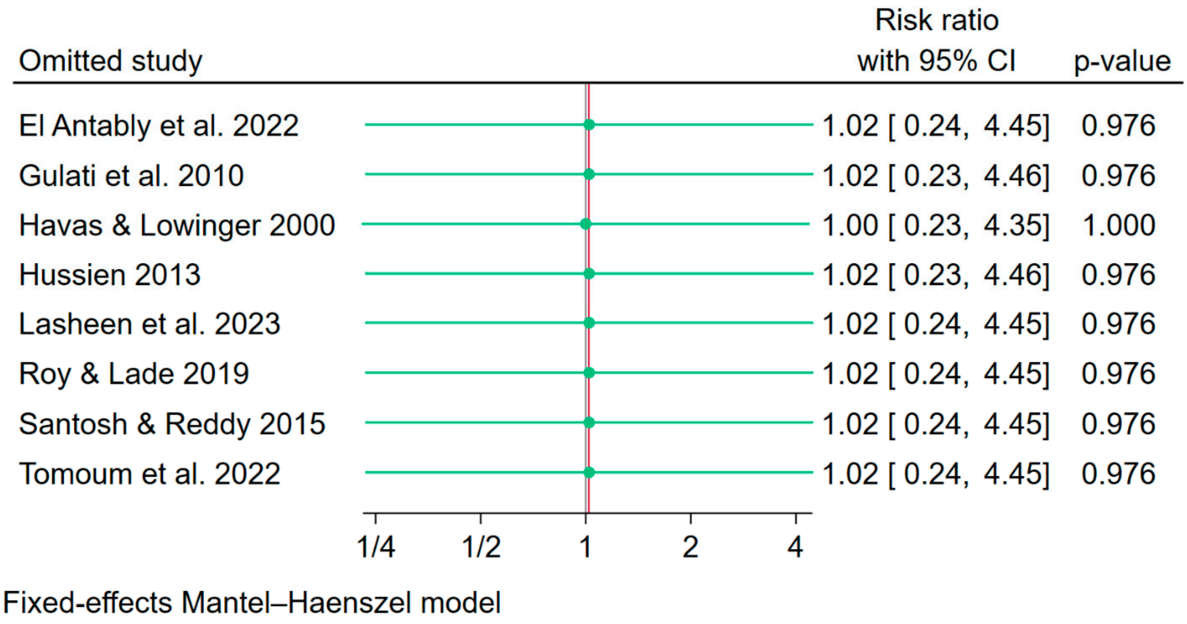

FIGURE S7. Leave-one-out sensitivity analysis of postoperative middle meatus antrostomy obstruction rate.

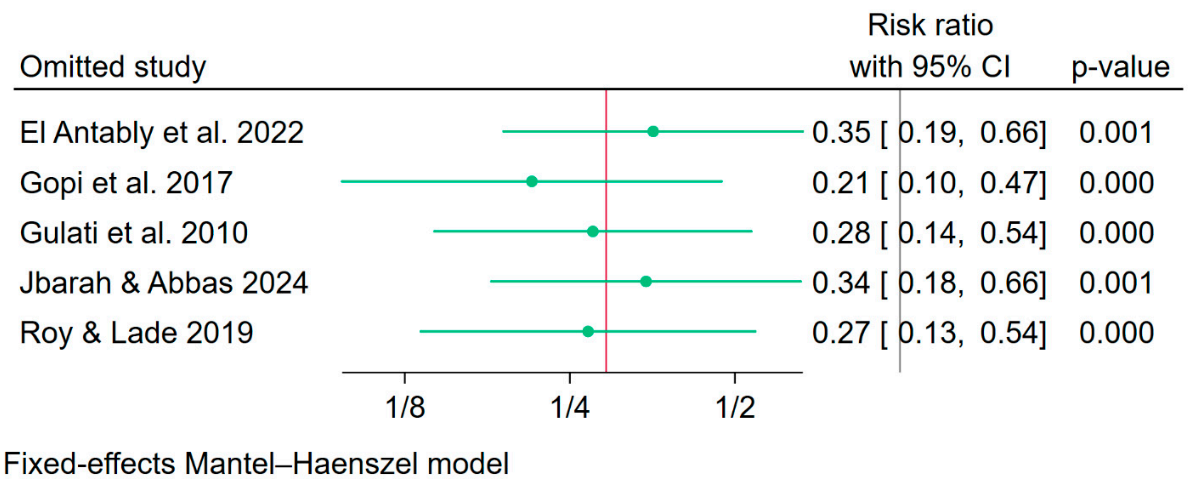

FIGURE S8. Leave-one-out sensitivity analysis of postoperative frontal recess obstruction rate.

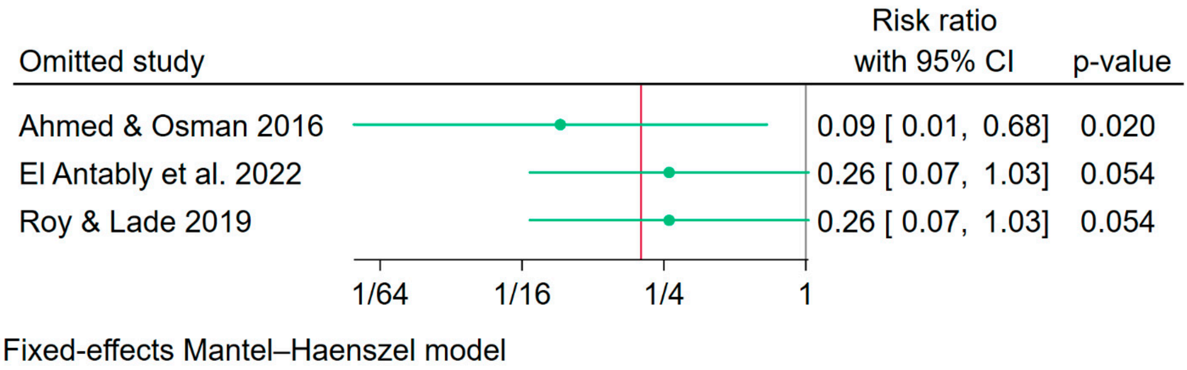

FIGURE S9. Leave-one-out sensitivity analysis of the mean smell test score.

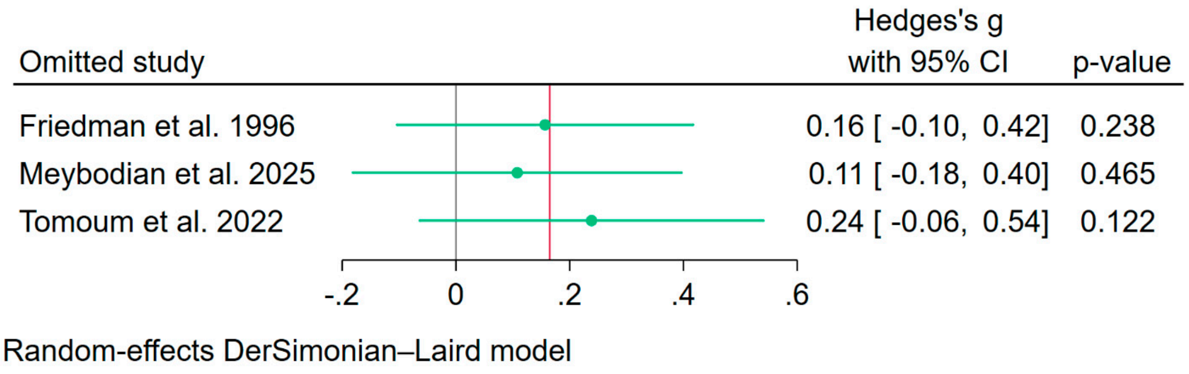

FIGURE S10. Leave-one-out sensitivity analysis of the mean SNOT score.

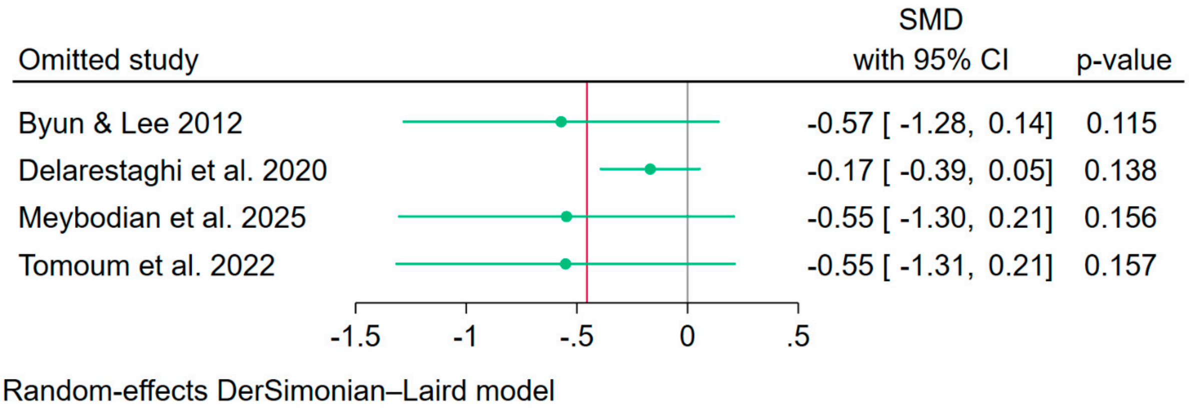

FIGURE S11. Leave-one-out sensitivity analysis of postoperative nasal discharge rate.

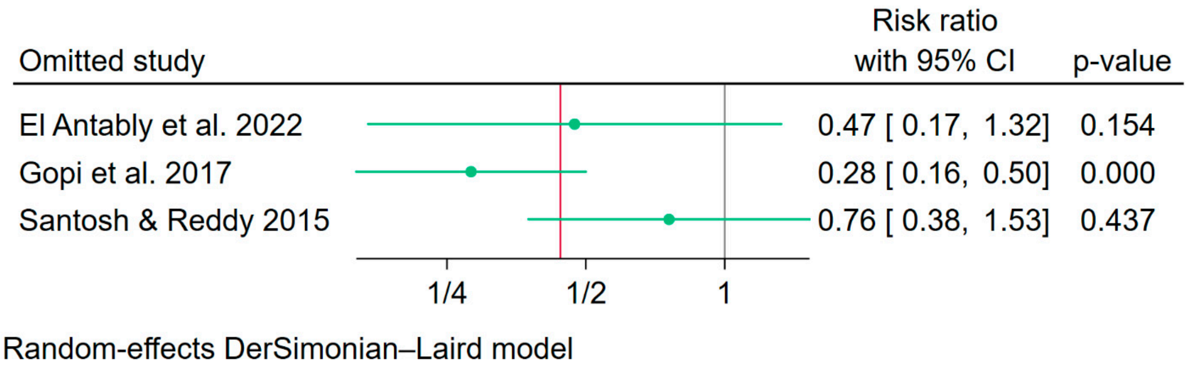

FIGURE S12. Leave-one-out sensitivity analysis of postoperative headache rate.

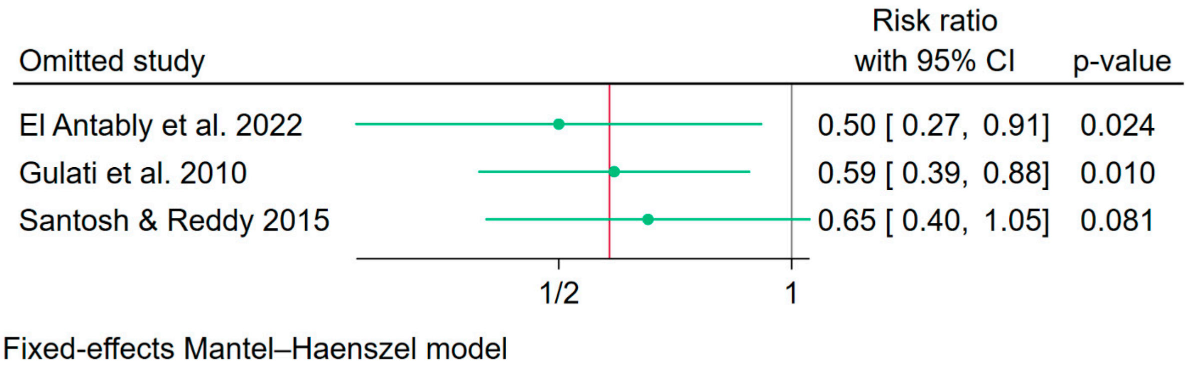

Supplement: Supplementary file 1 [file jcm-15-01288-s001.zip › jcm-4083170-supplementary.pdf]
